# Association between *SLC44A4*-*NOTCH4* SNPs and serum lipid levels in the Chinese Han and Maonan ethnic groups

**DOI:** 10.1186/s12986-020-00533-0

**Published:** 2020-12-14

**Authors:** Peng-Fei Zheng, Rui-Xing Yin, Yao-Zong Guan, Bi-Liu Wei, Chun-Xiao Liu, Guo-Xiong Deng

**Affiliations:** 1 Department of Cardiology, Institute of Cardiovascular Diseases, The First Affiliated Hospital, Guangxi Medical University, Nanning, 530021 Guangxi People’s Republic of China; 2 Guangxi Key Laboratory Base of Precision Medicine in Cardio-Cerebrovascular Disease Control and Prevention, Nanning, 530021 Guangxi People’s Republic of China; 3 Guangxi Clinical Research Center for Cardio-Cerebrovascular Diseases, Nanning, 530021 Guangxi People’s Republic of China

**Keywords:** Solute carrier family 44 member 4, Notch receptor 4, Single nucleotide polymorphism, rs577272, rs3134931, Haplotypes, Lipids

## Abstract

**Background:**

The current research was to assess the relationship of the solute carrier family 44 member 4 (*SLC44A4*) rs577272, notch receptor 4 (*NOTCH4*) rs3134931 SNPs and serum lipid levels in the Han and Maonan ethnic groups.

**Methods:**

The genetic makeup of the *SLC44A4* rs577272 and *NOTCH4* rs3134931 SNPs in 2467 unrelated subjects (Han, 1254; Maonan,1213) was obtained by using polymerase chain reaction and restriction fragment length polymorphism technique, combined with gel electrophoresis, and confirmed by direct sequencing.

**Results:**

The genotype frequencies of *SLC44A4* rs577272 and *NOTCH4* rs3134931 SNPs were different between Han and Maonan populations (*P* < 0.05); respectively. The *SLC44A4* rs577272 SNP was associated with total cholesterol (TC) and high-density lipoprotein cholesterol (HDL-C) levels in Maonan group. The *NOTCH4* rs3134931 SNP was associated with triglyceride (TG) in Han; and TG and low-density lipoprotein cholesterol (LDL-C) levels in Maonan groups (*P* < 0.025–0.001). Stratified analysis according to gender showed that the *SLC44A4* rs577272 SNP was associated with TC and HDL-C in Han and Maonan females; TC in Maonan males, meanwhile, the *NOTCH4* rs3134931 SNP was associated with TG and HDL-C in Han males; TG in Han females; TG and LDL-C in Maonan males; and TG, HDL-C and LDL-C in Maonan females. Linkage disequilibrium analysis showed that the most common haplotype was rs577272G-rs3134931A (> 50%) in both Han and Maonan groups. The haplotype of rs577272G-rs3134931A was associated with TG and HDL-C in Han; and TC, TG and HDL-C in Maonan ethnic groups.

**Conclusions:**

These results suggest that the relationship among *SLC44A4* rs577272, *NOTCH4* rs3134931 SNPs and serum lipid parameters may vary depending on the gender and/or ethnicity/race in some populations. Haplotypes could explain more changes in serum lipid parameters than any single SNP alone particularly for TC, TG and HDL-C.

## Background

Dyslipidemia is heritable risk factor of coronary heart disease (CHD), which has been a prominent reason of disability, mortality, morbidity, functional deterioration and expensive healthcare, and accounts for approximately 30% of all the deaths worldwide [1–4]. Previous studies have shown that CHD occurs due to various factors and can be subjective to genomic background, lifestyle, environmental factors and alterations of plasma lipid levels as well as their interactions with each other [5, 6]. Coronary atherosclerosis is generally considered to be the pathological foundation of CHD [7], which is caused by the accumulation of cholesterol in arterial wall macrophages and the dysregulation of metabolic rate of lipids for example increased levels of total cholesterol (TC) [8], triglyceride (TG) [9], low-density lipoprotein cholesterol (LDL-C) [10], and apolipoprotein (Apo) B [11], along with reduced levels of ApoA1 [11] and high-density lipoprotein cholesterol (HDL-C) [12] in serum. Thus, it can be seen that hyperlipidaemia (HLP) acts as a crucial risk factor for CHD and its complications. HLP is deemed to be affected by various hereditary and environmental elements and their connections [13].

Previous genome-wide association studies (GWASes) have demonstrated that the rs577272 SNP near the Solute carrier family 44 member 4 gene (*SLC44A4*; also knows as: *CTL4*; *NG22*; *TPPT*; *DFNA72*; *hTPPT1*; *C6orf29*, GeneID:80736, HGNC ID: 13941, locus type: gene with protein product, located in chromosome 6p21.33) was associated with serum TC and C-reactive protein (CRP) levels, which are all risk factors for CHD [14]. At the same time, the rs3134931 SNP near the neurogenic locus notch homolog protein 4 gene (*NOTCH4*; also knows as: *INT3*, Gene ID: 4855, HGNC ID: 7884, locus type: gene with protein product, located in chromosome 6p21.32) may result in regulating serum myeloperoxidase (MPO) levels in Europeans [15]. Some researchers have demonstrated that serum levels of MPO are linked with the elevated risk of CHD by a mechanism inducing dysfunctional HDL particles [16] and MPO-dependent LDL oxidation [17]. Previous work has also demonstrated that endothelial *NOTCH* signaling is impacted by lipid-mediated inflammatory status, and its down-regulation seems to correlate with an inflammatory state in the endothelium, and all *NOTCH* receptors (*NOTCH*1-4) are expressed in the vascular system [18]. It is noticeable that *NOTCH4* expression is significantly reduced in patients with HLP, *NOTCH4* is a pathogenic factor involved in the process that lipids lead to vascular endothelial inflammation [19]. Nevertheless, the association among the *SLC44A4* rs577272, *NOTCH4* rs3134931 SNPs and serum lipid levels in Han and Maonan ethnic groups is not clear and not reported in literature.

China is well-known as a country with multiple ethnicities- that is composed of the Han nationality and 55 ethnic minorities. As per the sixth national census statistics of China (2010), the total population of the Maonan ethnic group was 107,166 (37th). Most of the Maonan people are located in Huanjiang Maonan Autonomous County, Guangxi Zhuang Autonomous Region. Although the population of Maonan is small, there are various differences in lifestyle and dietary habits between Maonan and local Han populations, the marriage custom in Maonan is relatively conservative. Maonan still maintain the custom of intra-ethnic marriages, thus, intermarriage with other ethnic groups is very rare [20]. Therefore, there was less diversity about their genetic background in Maonan population. As far as we know there has not been any previous study on the relationship among the *SLC44A4* rs577272, *NOTCH4* rs3134931 SNPs and serum lipid levels in the Han and Maonan ethnic groups. Thus, this study was designed to understand the relationship of the *SLC44A4* rs577272, *NOTCH4* rs3134931 SNPs and several environmental aspects with serum lipid levels in the Han and Maonan ethnic groups.

## Materials and methods

### Study populations

A total of 1254 (569 males, 45.37%; 685 females, 54.63%) unrelated participants of Han nationality and 1213 unrelated subjects (505 males, 41.63%; 708 females, 58.37%) of Maonan nationality were arbitrarily chosen based on our previously stratified randomized samples. All of the subjects were farm workers. They were staying in Huanjiang Maonan Autonomous County, Guangxi Zhuang Autonomous Region of China. They were in the age range of 16–88 years. There was not any difference in age distribution (57.58 ± 12.94 vs. 57.20 ± 15.08) and gender ratio between Han and Maonan groups, respectively. The selection criteria for Maonan individuals have been described in detail in our previous epidemiological studies [21, 22]. All subjects were basically healthy and none of them had a history of CHD, myocardial infarction (MI), ischemic stroke (IS) and type 2 diabetes mellitus (T2DM). They were not taking any medicines that could alter the lipid levels of serum. Before the beginning of the study, all participants had provided written informed consent. The study protocol was approved by the Ethics Committee of the First Affiliated Hospital, Guangxi Medical University (No. Lunshen-2014 KY-Guoji-001, Mar. 7, 2014).

### Epidemiological analysis

Universally standardized methods and protocols were used to conduct the epidemiological survey [23]. By using a standard set of questionnaires, details regarding lifestyle as well as demographic factors were collected. Alcohol consumption (0 (non-drinker), < 25 g/day and ≥ 25 g/day) and smoking status (0 (non-smoker), < 20 cigarettes/day and ≥ 20 cigarettes/day) were divided into three different subgroups. Current smoking was defined as more than one cigarette per day. The subjects who reported having smoked ≥ 100 cigarettes during their lifetime were classified as current smokers if they currently smoked and former smokers if they did not [21, 22]. As per the methods in previously published studies, the weight, height, body mass index (BMI, kg/m^2^), blood pressure, and waist circumference were measured [24].

### Biochemical assays

A fasting venous blood sample (5 mL) was collected from each participant. A part of the sample (2 mL) was collected into glass tubes to measure serum lipid levels. The remaining 3 mL of the sample was collected in the tubes containing anticoagulants (13.20 g/L tri-sodium citrate, 4.80 g/L citric acid, and 14.70 g/L glucose) and was utilized to extract deoxyribonucleic acid (DNA). Measurements of serum TG, TC, LDL-C, and HDL-C levels in the samples were performed by enzymatic methods with commercially available kits (RANDOX Laboratories Ltd., Ardmore, Diamond Road, Crumlin Co. Antrim, United Kingdom, BT29 4QY; Daiichi Pure Chemicals Co., Ltd., Tokyo, Japan). Serum ApoA1 and ApoB levels were detected by the immunoturbidimetric immunoassay using a commercial kit (APO CAL; cat. no. LP3023; Randox Laboratories, Ltd) [25]. Fasting blood glucose was determined with a glucose meter (Accu-Chek; F. Hoffman-La Roche AG, Basel, Switzerland). The values of serum lipid levels were tested by using an autoanalyzer (Type 7170A; Hitachi Ltd., Tokyo, Japan) in the Clinical Science Experiment Center of the First Affiliated Hospital, Guangxi Medical University [26, 27].

### Amplification of DNA and genotyping

The phenol–chloroform method was used to isolate genomic DNA from the peripheral blood leucocytes of the blood samples [28, 29]. The extracted DNA samples were stored at 4 °C till further use. PCR–RFLP was used to determine the *SLC44A4* rs577272 and *NOTCH4* rs3134931 SNP genotypes. The primer sequences of the *SLC44A4* rs577272 and *NOTCH4* rs3134931 SNPs as follows: forward 5′-ACTGTAGGTGCTCACTGGAT-3′ and reversed 5′-GATTCGTATTGCCATCGCCC-3′; forward 5′-AGAAGAGGAAAGGTGGAGGC-3′ and reversed 5′-AAGCTGGGTGTCAATGGAGA-3′ (Sangon, Shanghai, People’s Republic of China); respectively. The PCR reaction mixture (final volume: 25 µL) contained 2.0 μL of genomic DNA, 1.0 μL of each primer (10 μmol/L), 12.5 μL of 2 × *Taq* PCR Master Mix (constituent: 0.1 U *Taq* polymerase/μL, 500 μM dNTP each and PCR buffer, Tiangen, Beijing, People’s Republic of China.), and 8.5 μL of DNase/RNase-free ddH_2_O. The cycle details for the reaction are as follows: 95 °C for 5 min, 95 °C for 30 s for denaturing, 59 °C for 30 s for annealing, and elongation for 35 s at 72 °C for 35 cycles. The final extension of 72 °C for 7 min was used to finish amplification. Electrophoresis was done by using 2.0% agarose gels to run PCR products and bands were visualized by using ultraviolet light (Universal Hood II; Bio-Rad Laboratories, Inc., Hercules, CA, USA), and the PCR products located in 522- and 490-bp bands represent the target genes. The restriction enzyme reaction system includes 5.0 μL amplified DNA, 8.8 μL nuclease-free water, 1.0 μL of 10 × buffer solution and 0.2 μL *Rsa*I restriction enzyme in a total volume of 15 µL digested at 37 °C overnight. Restriction enzyme was used to digest the amplified DNA. Next, the genotypes were recognized by running an electrophoresis with 2.0% agarose gel and were visualized under ultraviolet light (Universal Hood II; Bio-Rad Laboratories, Inc., Hercules, CA, USA). An experienced reader who was unaware of the epidemiological data and lipid levels scored genotypes. Different bands of enzyme-digested products represent different genotypes of *SLC44A4* rs577272 polymorphism (AA genotype, 522-bp; GA genotype, 522-, 448- and 74-bp; GG genotype, 448- and 74-bp); *NOTCH4* rs3134931 polymorphism (AA genotype, 490 bp; AG genotype 490-, 306- and 184-bp; GG genotype, 306- and 184-bp). Six samples detected by PCR–RFLP were also established by direct sequencing with an ABI Prism 3100 (Applied Biosystems, Shanghai Sangon Biological Engineering Technology & Services Co. Ltd., China).

### Analytical measures

Serum ApoB (0.80–1.05 g/L), TG (0.56–1.70 mmol/L), LDL-C (2.70–3.10 mmol/L), TC (3.10–5.17 mmol/L), HDL-C (1.16–1.42 mmol/L), ApoA1 (1.20–1.60 g/L) levels and the ApoA1/ApoB ratio (1.00–2.50) were defined as normal values at our Clinical Science Experiment Center. The participants with TC > 5.17 mmol/L and/or TG > 1.70 mmol/L were defined as HLP [30]. The diagnostic criteria of hypertension [31] and diabetes [32], overweight, normal weight, obesity [33] were also referred to previous studies.

### Statistical analyses

All data were evaluated by using SPSS (Version 22.0). The values of quantitative variables were presented as mean ± SD. Only serum TG levels were reported as medians and interquartile ranges. Direct counting was used to determine allele frequency. The standard goodness-of-fit test was utilized to verify the Hardy–Weinberg equilibrium (HWE). Chi-square test was used to assess the differences in the genotype distribution of selected 2 SNPs, the proportion of smokers and alcohol consumption between the two populations. The difference in general characteristics between Han and Maonan was analyzed by the independent-samples *t* test. Covariance analysis (ANCOVA) was used to test the relationship between blood lipid parameters and genotypes, and *P* < 0.025 (equivalent to *P* < 0.05 after adjusting for 2 SNPs independent tests by Bonferroni correction) was considered significantly statistical significance. The correlation between haplotypes/genotypes and the occurrence of HLP was detected by unconditional logistic regression analysis. Age, gender, BMI, alcohol consumption, cigarette smoking, and blood pressure were adapted for the statistical analysis. In order to estimate the link between the genotypes and some environmental elements with blood lipid levels in males and females of Han and Maonan populations, multivariable linear regression analysis with stepwise modeling was used. *P* value of < 0.05 was considered as statistically significant. Interactive heat map with several parameters related to blood lipid levels was drawn by R software (version 3.3.0) [34].

## Results

### General and biochemical characteristics

As mentioned in Table 1, the ApoA1/ApoB ratio, HDL-C and ApoA1 levels, were greater in Han than in Maonan nationalities (*P* < 0.05). The levels of serum TG, TC, LDL-C and ApoB, systolic and diastolic blood pressure, pulse pressure, the proportion of smokers and alcohol consumption were lesser in the Han than in the Maonan nationalities (*P* < 0.05–0.001). There was no obvious difference in age distribution, gender, height, BMI, weight, waist circumference and glucose between Han and Maonan nationalities. Subgroup analysis also found that the levels of ApoB, TC, weight, glucose, BMI, waist circumference, TG, systolic blood pressure, LDL-C, the proportion of smokers, diastolic blood pressure, alcohol consumption and pulse pressure were higher in HLP than in normal subjects in both Han and Maonan groups; the levels of ApoA1, HDL-C and the ApoA1/ApoB ratio were less in HLP than in normal subjects in both Han and Maonan groups; there was no any obvious difference in following factors such as gender, height, and age distribution in HLP than in normal subjects in both Han and Maonan groups.

**Table 1:** Comparison of demographic, lifestyle characteristics and serum lipid levels between the Han and Maonan populations

Parameter	Han			Maonan			*P*_Han versus Maonan_	*P*_Han_	*P*_Maonan_
Group	All	Normal	HLP	All	Normal	HLP			
Number	1254	662	592	1213	577	636			
Male/female^c^	569/685	295/367	274/318	505/708	231/346	274/362	0.061	0.541	0.282
Age (years)^a^	57.58 ± 12.94	57.32 ± 13.10	57.86 ± 11.53	57.02 ± 15.08	57.19 ± 14.31	57.76 ± 13.84	0.314	0.468	0.374
Height (cm)^a^	153.68 ± 7.56	153.46 ± 7.53	154.09 ± 7.59	153.55 ± 8.03	153.29 ± 7.84	153.78 ± 8.19	0.678	0.161	0.293
Weight (kg)^a^	52.57 ± 8.83	52.03 ± 8.27	54.27 ± 9.76	52.65 ± 10.89	51.48 ± 11.09	53.97 ± 10.65	0.954	5.38E−5	7.43E−5
Body mass index (kg/m^2^)^a^	22.35 ± 3.43	22.12 ± 3.44	22.80 ± 3.38	22.26 ± 3.76	21.80 ± 4.05	22.71 ± 3.43	0.605	0.001	3.23E–5
Waist circumference^a^	75.56 ± 7.89	74.56 ± 7.42	77.45 ± 8.40	75.95 ± 9.36	74.16 ± 9.25	77.58 ± 9.16	0.261	4.84E−10	1.53E−10
Smoking status [n (%)]^c^									
Non-smoker	997 (79.47)	561 (84.74)	436 (73.65)	901 (73.12)	447 (75.04)	454 (71.38)			
≤ 20 cigarettes/day	227 (18.13)	85 (12.84)	142 (21.45)	271 (23.50)	110 (21.49)	161 (25.31)			
20 cigarettes/day	30 (2.39)	16 (2.42)	14 (2.36)	41 (3.38)	20 (3.47)	21 (3.31)	0.008	1.76E-6	0.033
Alcohol consumption [n (%)]^c^									
Non-drinker	1040 (82.93)	548 (82.78)	492 (83.11)	952 (78.48)	487 (80.94)	465 (76.26)			
≤ 25 g/day	106 (8.45)	83 (12.54)	23 (3.89)	140 (11.54)	47 (9.88)	93 (13.05)			
> 25 g/day	108 (8.61)	31 (4.68)	77 (13.00)	121 (9.98)	43 (9.18)	78 (10.69)	0.013	3.38E−12	1.05E−5
Systolic blood pressure (mmHg)^a^	129.86 ± 19.68	118.61 ± 11.03	151.04 ± 14.15	135.37 ± 23.84	133.56 ± 24.60	137.01 ± 23.03	4.35E−10	2.53E−262	0.012
Diastolic blood pressure (mmHg)^a^	79.22 ± 11.66	74.24 ± 7.67	88.60 ± 12.10	83.00 ± 12.26	82.55 ± 12.77	83.42 ± 11.79	5.53E−15	8.68E−117	0.217
Pulse pressure (mmHg)^a^	50.64 ± 15.57	44.37 ± 9.05	62.44 ± 14.22	52.36 ± 16.93	51.00 ± 16.70	53.59 ± 17.06	0.009	4.01E−101	0.008
Glucose (mmol/L)^a^	6.03 ± 1.63	5.91 ± 1.65	6.24 ± 1.57	6.14 ± 1.43	5.93 ± 1.54	6.15 ± 1.39	0.061	0.001	0.027
Total cholesterol (mmol/L)^a^	4.82 ± 1.06	4.68 ± 1.04	5.09 ± 1.05	4.98 ± 1.06	4.38 ± 1.06	5.52 ± 1.10	2.95E−4	1.39E−10	1.14E−93
Triglyceride (mmol/L)^b^	1.00 (0.80)	0.87 (0.45)	1.43 (1.36)	1.08 (0.82)	0.93 (0.51)	1.37 (1.24)	0.021	2.18E−36	8.10E−35
HDL-C (mmol/L)^a^	1.73 ± 0.53	1.75 ± 0.59	1.67 ± .42	1.55 ± 0.48	1.66 ± 0.44	1.54 ± 0.51	1.92E−17	0.010	6.21E−5
LDL-C (mmol/L)^a^	2.73 ± 0.87	2.65 ± 0.84	2.88 ± 0.90	2.80 ± 0.82	2.45 ± 0.52	3.11 ± 0.91	0.045	3.25E−6	1.42E−48
ApoA1 (g/L)^a^	1.46 ± 0.31	1.48 ± 0.28	1.35 ± 0.29	1.38 ± 0.29	1.41 ± 0.35	1.30 ± 0.23	6.71E−8	2.04E−5	0.003
ApoB (g/L)^a^	0.83 ± 0.21	0.81 ± 0.20	0.88 ± 0.23	0.88 ± 0.23	0.78 ± 0.16	0.97 ± 0.23	4.43E−7	1.59E−8	2.10E−52
ApoA1/ApoB^a^	1.83 ± 0.59	1.85 ± 0.53	1.78 ± 0.68	1.68 ± 0.63	1.80 ± 0.57	1.57 ± 0.66	1.36E−9	0.028	4.84E−11

*HDL-C* high-density lipoprotein cholesterol, *LDL-C* low-density lipoprotein cholesterol, *Apo* apolipoprotein, *HLP* hyperlipidaemia. The value of triglyceride was presented as median (interquartile range) for not a normal distribution

^a^Mean ± SD determined by *t* test

^b^Median (interquartile range) tested by the Wilcoxon–Mann–Whitney test

^c^The rate or constituent ratio between the different groups was analyzed by the chi-square test

### Results of electrophoresis and genotyping

**Fig. 1:**
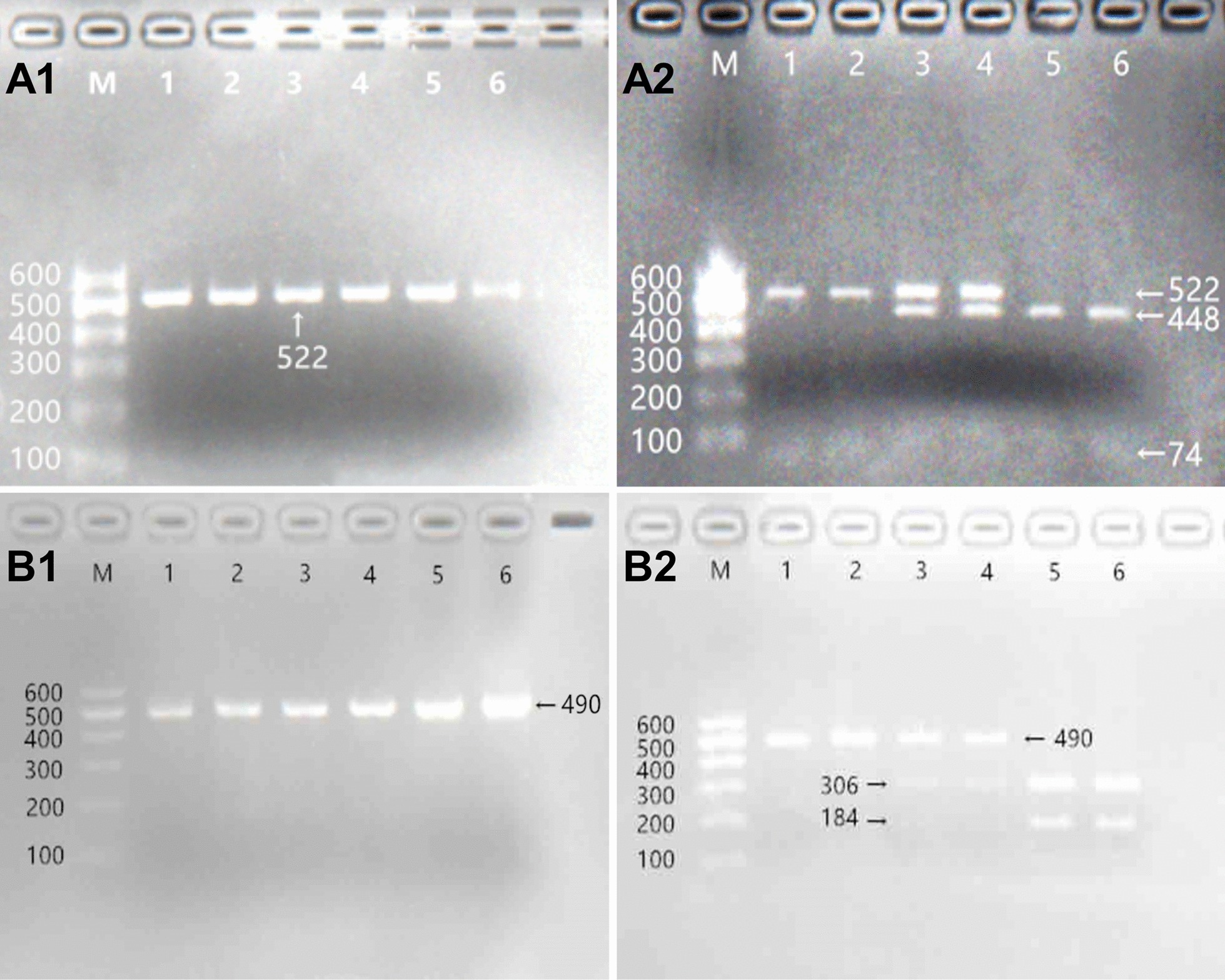
Agarose gel electrophoresis (2%) of PCR products and genotyping of the *SLC44A4* rs577272 and *NOTCH4* rs3134931 SNPs. **a1, a2** (rs577272): Lane M is the 100 bp marker ladder; Lanes 1–6 are samples, the 522 bp is the target genes. lanes 1 and 2, AA genotype (522 bp); lanes 3 and 4, AG genotype (522-, 448- and 74-bp); and lanes 5 and 6, GG genotype (448- and 74-bp). **b1**, **b2** (rs3134931): Lane M is the 100 bp marker ladder; Lanes 1–6 are samples, the 490 bp is the target genes. lanes 1 and 2, GG genotype (490 bp); lanes 3 and 4, AG genotype (490-, 306- and 184-bp); and lanes 5 and 6, AA genotype (306- and 184-bp)

**Fig. 2:**
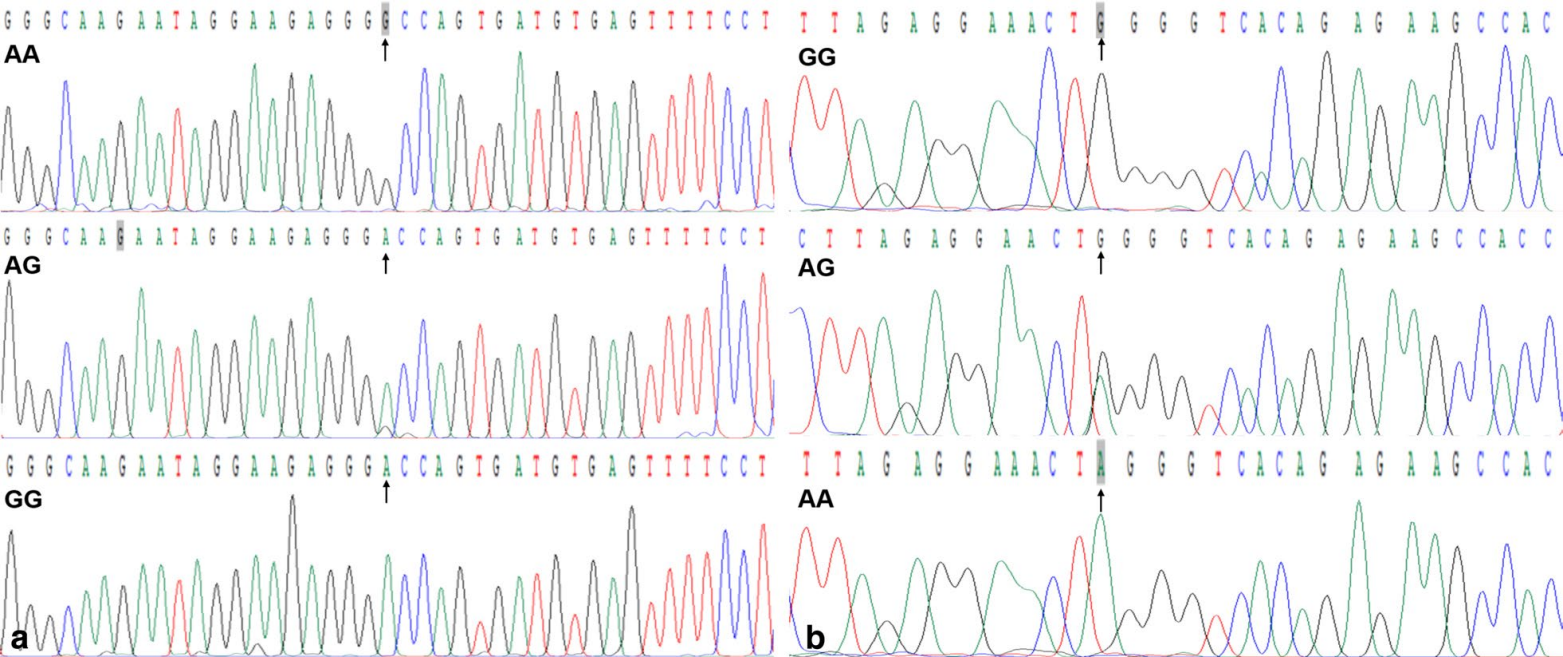
A part of the nucleotide sequence of the *SLC44A4* rs577272 (**a**) and *NOTCH4* rs3134931 SNPs (**b**)

Results from PCR and electrophoresis showed that each sample had the presence of 522-bp (Fig. 1a1) nucleotide sequences. The AA (522-bp), AG (522-, 448- and 74-bp) and GG (448- and 74-bp) genotypes of rs577272 SNP were shown in Fig. 1a2, respectively. The PCR product of the rs3134931 SNP was 490-bp nucleotide sequences (Fig. 1b1). The GG (490-bp), AG (490-, 306- and 184-bp) and AA (306- and 184-bp) genotypes were shown in Fig. 1b2, respectively. In addition, the genotypes of rs577272 and rs3134931 SNPs detected by PCR–RFLP were also verified by direct sequencing (Fig. 2).

### Genotypic and allelic frequencies and the connection with serum lipid levels and the risk of HLP

The genotypic scattering of the *SLC44A4* rs577272 and *NOTCH4* rs3134931 SNPs in both Han and Maonan populations conformed to HWE (*P* > 0.05). As shown in Table 2, the genotype frequencies of *SLC44A4* rs577272 and *NOTCH4* rs3134931 SNPs were different between Han and Maonan populations (*P* < 0.05); respectively. As shown in Table 3, the genotypes of the rs577272 SNP were associated with the risk of HLP in different genetic models: co-dominant model: GA versus AA (OR = 1.69, 95% CI = 1.27–2.24, *P* = 0.0011); dominant model: GA/GG versus AA (OR = 1.65, 95% CI = 1.25–2.17, *P* < 0.0001); overdominant model: AA/GG versus GA (OR = 1.60, 95% CI = 1.23–2.08, *P* < 0.0001) and log-additive model: G versus A (OR = 1.36, 95% CI = 1.09–1.71, *P* = 0.0067) in Maonan ethnic group. The genotypes of the rs3134931 SNP were associated with the risk of HLP in different genetic models: co-dominant model: AG versus GG (OR = 1.32, 95% CI = 0.98–1.78, *P* < 0.0001); dominant model: AG/AA versus GG (OR = 1.58, 95% CI = 1.19–2.09, *P* = 0.0014); recessive model: GG/AG versus AA (OR = 1.94, 95% CI = 1.46–2.57, *P* < 0.0001) and log-additive model: A versus G (OR = 1.53, 95% CI = 1.29–1.82, *P* < 0.0001) in Han ethnic group and co-dominant model: AG versus GG (OR = 1.46, 95% CI = 1.10–1.95, *P* = 0.0016); dominant model: AG/AA versus GG (OR = 1.57, 95% CI = 1.21–2.04, *P* < 0.0001); recessive model: GG/AG versus AA (OR = 1.45, 95% CI = 1.08–1.96, *P* < 0.014) and log-additive model: A versus G (OR = 1.35, 95% CI = 1.14–1.59, *P* < 0.0001) in Maonan ethnic group. As shown in Table 4, The *SLC44A4* rs577272 SNP was associated with TC and HDL-C in Maonan group, the *NOTCH4* rs3134931 SNP was associated with TG in Han; TG and LDL-C in Maonan group (*P* < 0.025–0.001). Stratified analysis according to gender showed that the *SLC44A4* rs577272 SNP was associated with TC in Maonan males; TC and HDL-C in Han and Maonan females; TC in Maonan males, meanwhile, the *NOTCH4* rs3134931 SNP was associated with TG and HDL-C in Han males; TG in Han females; TG and LDL-C in Maonan males; TG, HDL-C and LDL-C in Maonan females (*P* < 0.025–0.001).

**Table 2:** Genotypic and allelic frequencies of the two SNPs in the Han and Maonan ethnic groups [n (%)]

SNP	Genotype	Han			Maonan			*P*_Han versus Maonan_	*P*_Han_	*P*_Maoan_
		All (n = 1254)	Normal (n = 662)	HLP (n = 592)	All (n = 1213)	Normal (n = 577)	HLP (n = 636)			
*SLC44A4* rs577272 A>G	A/A	394 (31)	215 (32)	179 (30)	309 (25)	182 (32)	127 (20)			
	A/G	638 (51)	331 (50)	307 (52)	602 (50)	270 (47)	332 (52)			
	G/G	222 (18)	116 (18)	106 (18)	302 (25)	125 (22)	177 (28)	1.09E−5	0.692	1.43E−5
	G	1426 (57)	761 (57)	665 (56)	1220 (50)	634 (55)	586 (46)			
	A	1082 (43)	563 (43)	519 (44)	1206 (50)	520 (45)	686 (54)	3.73E−6	0.508	1.28E−5
	*P*_HWE_	0.21	0.58	0.21	0.82	0.21	0.23			
*NOTCH4* rs3134931 G>A	G/G	315 (25)	183 (28)	132 (22)	352 (29)	200 (35)	152 (24)			
	A/G	625 (50)	344 (52)	281 (47)	595 (49)	267 (46)	328 (52)			
	AA	314 (25)	135 (20)	179 (30)	266 (22)	100 (19)	156 (25)	0.048	2.12E−4	2.54E−5
	G	1255 (50)	710 (54)	545 (46)	1299 (54)	667 (58)	632 (50)			
	A	1253 (50)	614 (46)	639 (54)	1127 (46)	487 (42)	640 (50)	0.014	1.46E-4	6.29E-5
	*P*_HWE_	0.27	0.49	0.23	0.64	0.23	0.48			

*P* value defined as Chi-square test probability

*SLC44A4* the synaptotagmin like 3 gene, *NOTCH4* the solute carrier family 22 member 3 gene, *HLP* hyperlipidaemia, *HWE* Hardy–Weinberg equilibrium

**Table 3:** Risk for gene models in each SNP between the normal and HLPpopulations

SNP	Model	Genotype		Han		Maonan	
		Reference	Effect	OR (95% CI)	*P*	OR (95% CI)	*P*
rs577272 A>G	Co-dominant	A/A	G/A	0.90 (0.68–1.18)	0.36	1.69 (1.27–2.24)	0.0011
			G/G	0.77 (0.54–1.10)		1.31 (0.77–2.22)	
	Dominant	A/A	G/A + G/G	0.86 (0.66–1.12)	0.27	1.65 (1.25–2.17)	4E−04
	Recessive	A/A + G/A	G/G	0.82 (0.60–1.13)	0.23	0.91 (0.56–1.48)	0.7
	Overdominant	A/A + G/G	G/A	0.99 (0.77–1.26)	0.92	1.60 (1.23–2.08)	4E−04
	Log-additive			0.88 (0.74–1.05)	0.16	1.36 (1.09–1.71)	0.0067
rs3134931 G>A	Co-dominant	G/G	A/G	1.32 (0.98–1.78)	< 0.0001	1.46 (1.10–1.95)	0.0016
			A/A	2.33 (1.65–3.29)		1.79 (1.27–2.50)	
	Dominant	G/G	A/G + A/A	1.58 (1.19–2.09)	0.0014	1.57 (1.21–2.04)	7E−04
	Recessive	G/G + A/G	A/A	1.94 (1.46–2.57)	< 0.0001	1.45 (1.08–1.96)	0.014
	Overdominant	G/G + A/A	A/G	0.86 (0.68–1.10)	0.24	1.17 (0.91–1.50)	0.24
	Log-additive			1.53 (1.29–1.82)	< 0.0001	1.35 (1.14–1.59)	4E−04

*P* value defined as Logistic test probability

*OR* odds ratio, *CI* confidence interval

**Table 4:** Comparison of the genotypes and serum lipid levels in the Han and Maonan populations

Genotype	n	TC (mmol/L)	TG (mmol/L)	HDL-C (mmol/L)	LDL-C (mmol/L)	ApoA1 (g/L)	ApoB (g/L)	ApoA1 /ApoB
SLC44A4 rs577272								
Han								
AA	394	4.75 ± 1.02	0.98(0.76)	1.71 ± 0.45	2.70 ± 0.93	1.43 ± 0.27	0.84 ± 0.24	1.81 ± 0.50
AG + GG	860	4.86 ± 0.98	1.06 (0.83)	1.73 ± 0.57	2.74 ± 0.84	1.44 ± 0.29	0.83 ± 0.20	1.83 ± 0.62
*F*		5.650	− 0.601	1.527	2.674	1.320	1.482	2.873
*P*		0.077	0.448	0.539	0.449	0.779	0.728	0.424
Han/Male								
AA	117	4.95 ± 0.99	0.99 (0.83)	1.64 ± 0.43	2.78 ± 0.85	1.38 ± 0.22	0.80 ± 0.23	1.82 ± 0.51
AG + GG	442	5.15 ± 1.06	1.11 (0.78)	1.75 ± 0.53	2.92 ± 0.82	1.40 ± 0.26	0.80 ± 0.20	1.84 ± 0.61
*F*		6.946	− 2.104	3.295	4.049	1.618	0.936	1.347
*P*		0.047	0.035	0.311	0.123	0.632	0.955	0.768
Han/Female								
AA	277	4.56 ± 1.15	0.92 (0.68)	1.84 ± 0.45	2.61 ± 0.96	1.53 ± 0.29	0.91 ± 0.26	1.83 ± 0.50
AG + GG	418	4.78 ± 1.01	1.04 (0.82)	1.69 ± 0.61	2.70 ± 0.83	1.49 ± 0.32	0.90 ± 0.28	1.80 ± 0.63
*F*		7.633	− 1.991	8.117	3.884	7.189	0.980	3.669
*P*		0.018	0.046	0.011	0.200	0.032	0.856	0.231
Maonan								
AA	309	4.80 ± 1.12	1.09 (0.78)	1.63 ± 0.56	2.83 ± 0.88	1.35 ± 0.27	0.86 ± 0.25	1.69 ± 0.53
AG + GG	904	5.03 ± 1.01	1.11 (0.89)	1.50 ± 0.45	2.85 ± 0.79	1.38 ± 0.31	0.90 ± 0.22	1.67 ± 0.66
*F*		7.760	− 0.274	7.746	1.515	2.958	3.173	1.525
*P*		0.012	0.784	0.015	0.682	0.381	0.318	0.679
Maonan/Male								
AA	46	4.70 ± 0.94	0.97 (0.57)	1.65 ± 0.41	2.72 ± 0.82	1.37 ± 0.30	0.85 ± 0.17	1.69 ± 0.46
AG + GG	419	4.94 ± 0.83	1.07 (0.76)	1.56 ± 0.43	2.80 ± 0.75	1.37 ± 0.37	0.87 ± 0.21	1.66 ± 0.65
*F*		8.088	− 1.032	3.800	3.569	0.925	1.506	3.862
*P*		0.004	0.302	0.205	0.256	0.963	0.553	0.226
Maonan/Female								
AA	263	4.87 ± 1.04	1.15 (0.90)	1.59 ± 0.68	2.89 ± 0.85	1.34 ± 0.25	0.87 ± 0.25	1.69 ± 0.50
AG + GG	485	5.08 ± 1.12	1.27 (1.04)	1.45 ± 0.72	2.95 ± 0.92	1.39 ± 0.24	0.92 ± 0.23	1.69 ± 0.64
*F*		7.717	− 1.159	7.901	3.085	7.246	6.432	0.945
*P*		0.015	0.246	0.013	0.352	0.030	0.055	0.923
NOTCH4 rs3134931								
Han								
GG	315	4.80 ± 1.05	0.86 (0.82)	1.77 ± 0.58	2.63 ± 0.86	1.47 ± 0.33	0.81 ± 0.21	1.83 ± 0.62
AG + AA	939	4.83 ± 1.07	1.26 (0.88)	1.71 ± 0.52	2.73 ± 0.87	1.42 ± 0.27	0.84 ± 0.21	1.79 ± 0.57
*F*		1.548	− 7.020	4.333	5.731	1.747	1.536	3.026
*P*		0.524	0.000	0.114	0.074	0.481	0.532	0.341
Han/Male								
GG	163	4.89 ± 0.97	0.80 (0.79)	1.71 ± 0.69	2.75 ± 0.86	1.35 ± 0.29	0.78 ± 0.21	1.81 ± 0.52
AG + AA	396	5.04 ± 1.04	1.10 (0.85)	1.54 ± 0.41	2.82 ± 0.79	1.39 ± 0.23	0.81 ± 0.21	1.83 ± 0.45
*F*		4.416	− 3.528	7.696	3.123	1.416	3.924	1.458
*P*		0.109	0.017	0.014	0.323	0.731	0.180	0.557
Han/Female								
GG	152	4.68 ± 1.12	0.98 (0.79)	1.83 ± 0.45	2.62 ± 0.90	1.60 ± 0.31	0.83 ± 0.22	1.90 ± 0.45
AG + AA	543	4.68 ± 1.06	1.30 (0.85)	1.87 ± 0.58	2.67 ± 0.88	1.48 ± 0.30	0.89 ± 0.21	1.73 ± 0.56
*F*		0.836	− 4.038	2.989	1.612	5.576	2.726	2.874
*P*		0.992	0.008	0.404	0.526	0.089	0.432	0.388
Maonan								
GG	352	4.90 ± 0.91	0.99 (0.86)	1.57 ± 0.43	2.76 ± 0.80	1.39 ± 0.25	0.84 ± 0.20	1.71 ± 0.48
AG + AA	861	5.03 ± 1.01	1.34 (0.84)	1.55 ± 0.50	2.92 ± 0.86	1.36 ± 0.31	0.90 ± 0.24	1.65 ± 0.57
*F*		7.035	− 4.444	1.097	7.241	3.914	2.676	1.422
*P*		0.041	0.000	0.815	0.022	0.186	0.443	0.742
Maonan/Male								
GG	78	4.82 ± 1.02	0.94 (0.73)	1.65 ± 0.45	2.59 ± 0.79	1.39 ± 0.27	0.83 ± 0.25	1.72 ± 0.45
AG + AA	387	4.93 ± 1.05	1.23 ± 0.81	1.62 ± 0.37	2.80 ± 0.83	1.37 ± 0.38	0.86 ± 0.34	1.70 ± 0.47
*F*		5.128	− 3.872	3.968	7.463	1.506	1.578	1.433
*P*		0.088	0.012	0.167	0.016	0.543	0.528	0.634
Maonan/Female								
GG	274	5.04 ± 1.11	1.05 (0.70)	1.52 ± 0.43	2.81 ± 0.72	1.39 ± 0.33	0.85 ± 0.23	1.70 ± 0.45
AG + AA	474	5.12 ± 1.20	1.40 (0.80)	1.40 ± 0.52	3.04 ± 0.80	1.36 ± 0.29	0.92 ± 0.30	1.63 ± 0.43
*F*		4.003	− 4.569	7.087	7.982	3.756	2.973	2.823
*P*		0.125	0.000	0.017	0.008	0.195	0.358	0.427

The value of triglyceride was presented as median (interquartile range) for not meet the normal distribution, the difference among the genotypes was determined by the Kruskal–Wallis test. The *P* value calculated by ANCOVA, using general linear models, and adjusted for age, sex, BMI, smoking status, alcohol use, glucose and hypertension, *P* < 0.025 was considered statistically significant (corresponding to *P* < 0.05 after adjusting for 2 independent tests by the Bonferroni correction). n = sample size

*TC* total cholesterol, *TG* triglyceride, *HDL-C* high-density lipoprotein cholesterol, *LDL-C* low-density lipoprotein cholesterol, *ApoA1* apolipoprotein A1, *ApoB* apolipoprotein B, *ApoA1/ApoB* the ratio of apolipoprotein A1 to apolipoprotein B

### Haplotype-based association with serum lipid levels and HLP

**Fig. 3:**
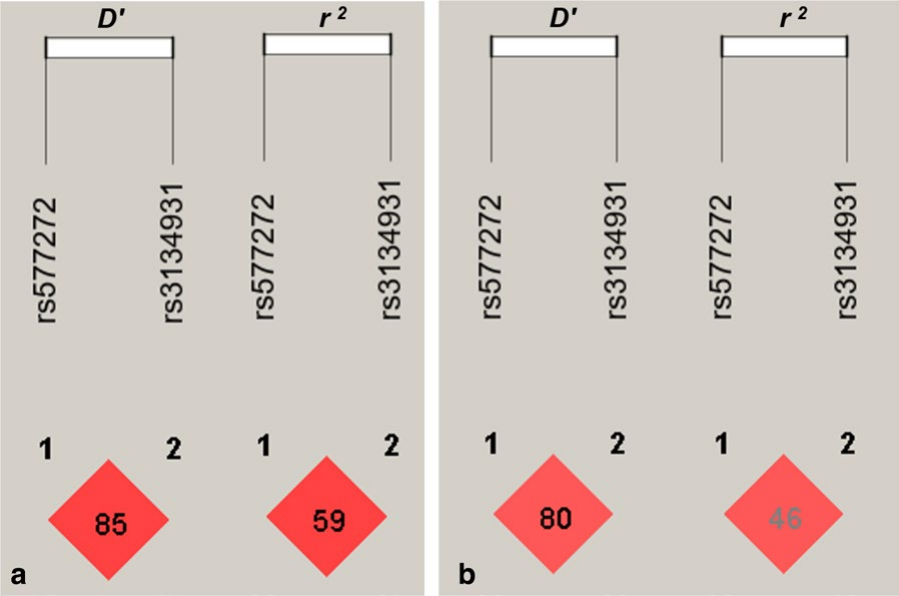
The linkage disequilibrium (LD) of the *SLC44A4* rs577272 and *NOTCH4* rs3134931 SNPs in the Han (**a**) and Maonan (**b**) groups

**Fig. 4:**
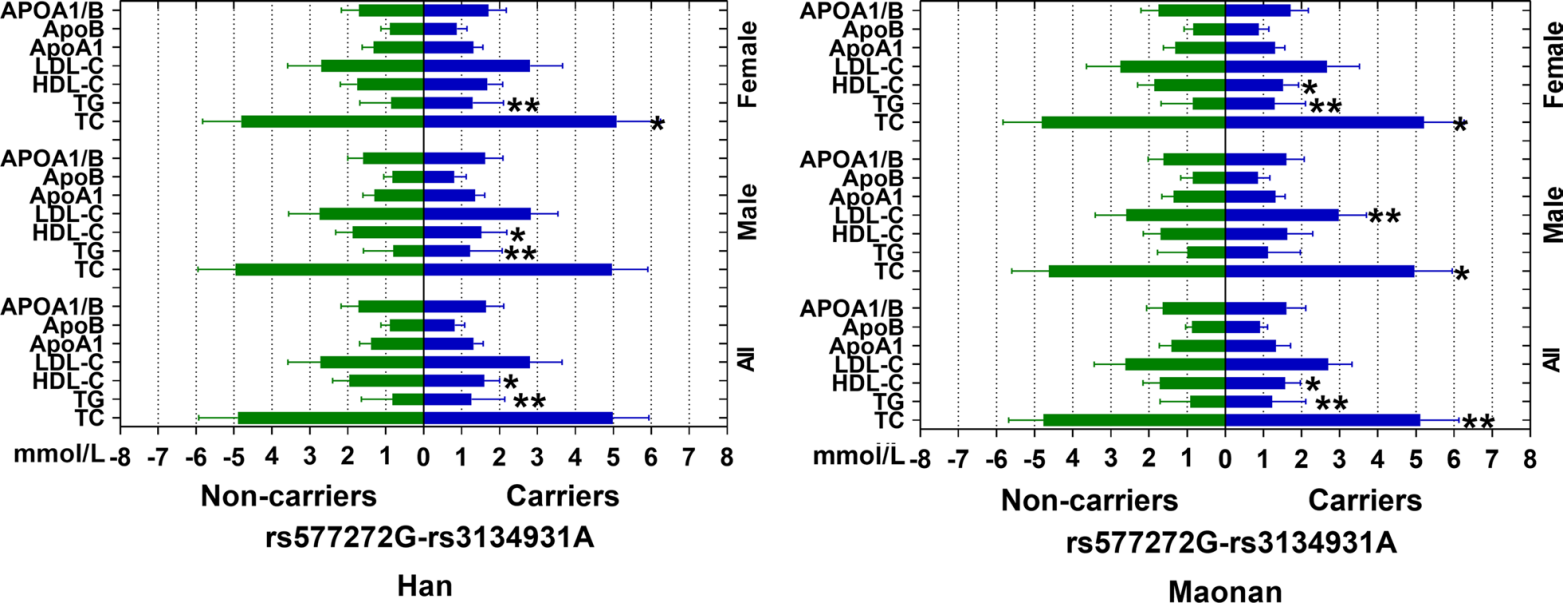
Lipid parameters according to the haplotypes of the Han and Maonan groups. *TC* total cholesterol, *TG* triglyceride, *HDL-C* high-density lipoprotein cholesterol, *LDL-C* low-density lipoprotein cholesterol, *Apo* apolipoprotein. ^*^P < 0.05; ^**^P < 0.001

Figure 3 indicates that there was strong pairwise linkage disequilibrium (LD) among the detected loci in both Han (A) and Maonan (B) groups. As shown in the Table 5, the dominant haplotype was the rs577272G-rs3134931A (> 50% of the samples). The haplotype of the rs577272G-rs3134931A was related to an increased morbidity of HLP in the both Han and Maonan groups, At the same time, Fig. 4 indicates that the haplotype of rs577272G-rs3134931A was associated with TG and HDL-C levels in Han; TC, TG and HDL-C levels in Maonan ethnic groups (*P* < 0.05–0.001, respectively). In addition, multivariate logistic analysis showed that the rs577272G-rs3134931A haplotype was positively correlated with the incidence of HLP in Han and Maonan according to stratified risk factors (gender, BMI, smoking, diabetes and blood pressure; Table 6).

**Table 5:** Association between the haplotypes among *SLC44A4* rs577272 SNP and *NOTCH4* rs3134931 SNP andHLP in the Han and Maonan group [n(frequency)]

No	Haplotypes	Han				Maonan			
		Normal	HLP	OR [95% CI]	*P* value	Normal	HLP	OR [95% CI]	*P* value
S1	rs577272A-rs3134931A	395.94 (0.284)	361.33 (0.260)	0.930 [0.868–1.221]	0.4376	239.27 (0.208)	273.27 (0.192)	0.914 [1.024–1.201]	0.7876
S2	rs577272A-rs3134931G	363.81 (0.261)	302.59 (0.218)	0.910 [0.758–1.082]	0.1756	392.64 (0.341)	373.69 (0.263)	0.849 [0.742–1.034]	0.1700
S3	rs577272G-rs3134931A	284.85 (0.204)	379.42 (0.275)	2.289 [2.017–2.620]	0.0021	251.94 (0.219)	453.62 (0.320)	2.442 [2.229–2.698]	0.0011
S4	rs577272G-rs3134931G	350.99 (0.251)	342.61 (0.247)	0.906 [0.741–1.026]	0.5580	267.15 (0.232)	319.23 (0.225)	0.917 [0.879–1.258]	0.6540

Rare Hap (frequency < 1%) in both populations has been dropped. *P* was obtained by unconditional logistic regression analysis

*HLP* hyperlipidaemia, *SLC44A4* solute carrier family 44 member 4, *NOTCH4* notch receptor 4

**Table 6:** The *SLC44A4* rs577272G-*NOTCH4* rs3134931A haplotype and HLP in the Han and Maonan populations according to stratified risk factors

Factor	Type	Haplotype	OR (95%CI) _Han_	*P*_Han_	OR (95%CI) _Maonan_	*P*_*Maonan*_
Gender	Male	G-A non-carriers	1	–	1	–
	Female	G-A carriers	1.629 (1.176–2.257)	0.003	1.721 (1.223–2.451)	0.023
BMI	< 24 kg/m^2^	G-A non-carriers	1	–	1	–
	≥ 24 kg/m	G-A carriers	3.163 (1.976–4.064)	1.61E−6	3.413 (3.117–3.867)	2.01E−6
Smoking	Nonsmoker	G-A non-carriers	1	–	1	–
	Smoker	G-A carriers	2.412 (1.862–2.871)	1.48E−5	2.651 (1.935–3.312)	2.20E−6
Drinking	Nondrinker	G-A non-carriers	1	–	1	–
	Drinker	G-A carriers	0.859 (0.635–1.163)	0.326	0.912 (0.712–1.221)	0.412
Diabetes	Non-diabetes	G-A non-carriers	1	–	1	–
	Diabetes	G-A carriers	2.802 (2.208–3.390)	2.52E−5	2.445 (2.037–3.088)	1.15E−5
Blood pressure	Normotensive	G-A non-carriers	1	–	1	–
	Hypertension	G-A carriers	2.234 (1.782–2.653)	6.12E−4	2.534 (1.982–2.853)	4.22E−4

### Relationship among lipid parameters and alleles/genotypes

Table 7 indicates that the association between serum lipid parameters and the alleles and/or genotypes of two selected SNPs in Han and Maonan groups. The results showed that the alleles of rs577272 were associated with TC and HDL-C in Han and Maonan ethnic groups; and the genotypes of rs577272 were associated with TC and HDL-C in Maonan ethnic group; the alleles of rs3134931 were associated with TC and HDL-C in Han enthic group and TG, HDL-C and LDL-C in Maonan ethnic group; the genotypes of rs3134931 were associated with TG in Han ethnic group and TG and LDL-C in Maonan ethnic group (*P* < 0.005–0.001); respectively.

**Table 7:** Correlation between serum lipid parameters and the *SLC44A4* rs577272 SNP and *NOTCH4* rs3134931 SNP alleles/genotypes in the Han and Maonan populations

Lipid	SNP	Allele	Genotype	Std.error	Beta	*t*	*P*
Han + Maonan							
TC	rs577272	A/G		0.009	− 0.027	− 3.169	0.002
	rs577272		AA/GA/GG	0.019	0.065	3.394	0.001
TG	rs3134931	G/A		0.068	− 0.236	− 3.495	4.92E−4
			GG/AG/AA	0.077	− 0.212	− 2.749	0.006
HDL-C	rs577272		AA/GA/GG	0.043	0.112	2.580	0.010
LDL-C	rs3134931		AA/GA/GG	0.021	− 0.071	− 3.395	0.001
Han							
TC	rs577272	A/G		0.065	0.117	2.717	0.007
TG	rs3134931	G/A		0.068	− 0.236	− 3.495	4.913E−4
			GG/AG/AA	0.019	0.065	3.394	0.001
HDL-C	rs3134931	G/A		0.091	− 0.359	− 3.958	7.95E−5
	rs577272	A/G		0.137	0.276	2.014	0.044
Maonan							
TC	rs577272	A/G		0.092	0.527	6.103	9.24E−10
	rs577272		AA/GA/GG	0.069	− 0.305	− 4.437	9.33E−6
TG	rs3134931	G/A		0.064	− 0.199	− 3.091	0.004
	rs3134931		AA/GA/GG	0.049	− 0.128	− 2.628	0.008
HDL-C	rs577272		AA/GA/GG	0.035	− 0.119	− 3.754	1.83E−4
	rs577272	A/G		0.058	− 0.16	− 2.725	0.006
	rs3134931	G/A		0.031	− 0.094	− 3.236	0.002
LDL-C	rs3134931		AA/GA/GG	0.093	0.567	6.123	9.04E−10
	rs3134931	G/A		0.069	− 0.305	− 4.417	4.83E−6

Association of serum lipid traits and allele and genotypes in Maonan, Han and combined the Maonan and Han populations were assessed by multivariable linear regression analyses with stepwise modeling

*TC* total cholesterol, *HDL-C* high-density lipoprotein cholesterol, *Apo* apolipoprotein, *Beta* standardized coefficient

### Correlated environment factors for serum lipid parameters

As shown in Tables 8 and 9, multivariable linear regression analysis showed that several environmental factors such as gender, age, glucose levels, waist circumference, BMI, systolic and diastolic blood pressure, pulse pressure, smoking and drinking were associated with serum lipid parameters in both ethnic groups or in males and females (*P* < 0.05–0.001 for all).

**Table 8:** Relationship between serum lipid parameters and relative factors in the Han and Maonan populations

Lipid	Risk factor	B	Std.error	Beta	*t*	*P*
Han and Maonan						
TC	Waist circumference	0.021	0.003	0.173	7.626	3.63E−4
	Diastolic blood pressure	0.010	0.002	0.117	5.290	1.35E−7
	Age	0.007	0.002	0.086	3.962	7.69E−5
	Height	− 0.013	0.003	− 0.094	− 3.923	9.02E−5
	Cigarette smoking	0.130	0.050	0.059	2.610	0.009
	Ethnic group	0.103	0.045	0.048	2.285	0.022
TG	Pulse pressure	− 0.004	0.001	0.072	− 3.311	0.001
	Cigarette smoking	− 0.116	0.056	0.055	− 2.068	0.039
	Height	− 0.010	0.003	− 0.093	− 3.596	3.30E−4
HDL-C	Ethnic group	− 0.194	0.023	− 0.176	− 8.325	1.49E−6
	Weight	0.005	0.001	0.090	− 3.921	9.10E−5
	Gender	0.089	0.026	0.079	3.420	0.001
LDL-C	Waist circumference	0.018	0.002	0.190	8.407	7.62E−17
	Alcohol consumption	− 0.209	0.033	− 0.156	− 6.379	2.19E−10
	Ethnic group	− 0.004	0.001	− 0.100	− 3.758	1.76E−4
ApoA1	Alcohol consumption	0.131	0.011	0.280	11.464	1.46E−9
	Cigarette smoking	0.117	0.016	0.192	7.441	1.44E−13
	Weight	− 0.002	0.001	− 0.074	− 2.269	0.023
	Waist circumference	− 0.002	0.001	− 0.074	− 2.399	0.017
ApoB	Waist circumference	0.007	0.001	0.294	13.301	7.78E−9
	Systolic blood pressure	0.001	0.000	0.100	4.735	2.34E−6
	Height	− 0.002	0.001	− 0.052	− 2.417	0.016
ApoA1/ApoB	Waist circumference	− 0.019	0.001	− 0.274	− 13.042	1.87E−37
	Alcohol consumption	0.160	0.024	0.166	6.711	2.47E−11
	Cigarette smoking	0.167	0.033	0.134	5.132	3.12E−7
Han						
TC	Diastolic blood pressure	0.017	0.003	0.192	6.289	4.70E−10
	Waist circumference	0.016	0.004	0.121	3.823	1.40E−4
	Glucose	0.056	0.019	0.087	2.919	0.004
	Gender	− 0.010	0.005	− 0.076	− 2.265	0.024
TG	Glucose	0.036	0.016	0.068	2.225	0.026
	Cigarette smoking	− 0.125	0.051	− 0.075	− 2.449	0.014
	Pulse pressure	− 0.007	0.002	− 0.138	− 4.510	7.21E−6
HDL-C	Weight	− 0.015	0.002	− 0.238	− 7.359	3.74E−4
	Alcohol consumption	0.097	0.028	0.113	3.501	4.83E−4
	Gender	− 0.005	0.002	− 0.098	− 2.538	0.011
LDL-C	Waist circumference	0.016	0.003	0.148	4.846	1.45E−6
	Systolic blood pressure	0.008	0.002	0.194	5.417	7.53E−8
	Age	− 0.006	0.001	− 0.147	− 4.213	2.73E−5
ApoA1	Alcohol consumption	0.189	0.014	0.423	13.732	1.38E−9
	Cigarette smoking	0.143	0.017	0.255	8.598	2.90E−7
	Weight	− 0.006	0.001	− 0.174	− 6.225	6.93E−10
ApoB	Waist circumference	0.006	0.001	0.224	5.731	1.30E−8
	Glucose	0.012	0.004	0.092	3.165	0.002
	BMI	0.005	0.002	0.085	2.227	0.026
ApoA1/ApoB	Waist circumference	− 0.013	0.003	− 0.168	− 3.744	1.91E−4
	Alcohol consumption	0.204	0.033	0.222	6.239	6.39E−10
	Cigarette smoking	0.204	0.043	0.176	4.798	1.84E−6
Maonan						
TC	Waist circumference	0.023	0.004	0.202	6.206	0.000
	Age	0.007	0.002	0.103	3.302	0.001
	Diastolic blood pressure	0.005	0.003	0.062	1.966	0.050
	Height	− 0.010	0.005	− 0.076	− 2.265	0.024
TG	Alcohol consumption	0.178	0.060	0.108	2.980	0.003
	Cigarette smoking	− 0.183	0.086	− 0.082	− 2.125	0.034
	Weight	− 0.015	0.002	− 0.238	− 7.359	3.74E−13
HDL-C	Gender	0.158	0.042	0.143	3.783	1.64E−4
	Systolic blood pressure	0.002	0.001	0.087	2.835	0.005
LDL-C	Alcohol consumption	− 0.310	0.039	− 0.231	− 7.920	5.92E−5
	Waist circumference	0.017	0.003	0.194	6.633	5.21E−5
	Age	0.008	0.002	0.140	4.856	1.38E−6
ApoA1	Waist circumference	− 0.004	0.001	− 0.143	− 4.708	2.83E−6
	Alcohol consumption	0.071	0.015	0.147	4.855	1.38E−6
	Glucose	0.014	0.06	0.067	− 2.227	0.026
ApoB	Waist circumference	0.007	0.001	0.297	9.806	8.51E−6
	Age	0.001	0.000	0.071	2.197	0.028
	Pulse pressure	0.001	0.000	0.070	2.133	0.033
ApoA1/ApoB	Waist circumference	− 0.017	0.002	− 0.261	− 8.784	6.16E−8
	Alcohol consumption	0.161	0.029	0.163	5.552	3.56E−8
	Pulse pressure	− 0.003	0.001	− 0.095	− 3.234	0.001

*TC* total cholesterol, *TG* triglyceride, *HDL-C* high-density lipoprotein cholesterol, *LDL-C* low-density lipoprotein cholesterol, *ApoA1* apolipoprotein A1, *ApoB* apolipoprotein B, *ApoA1/ApoB* the ratio of apolipoprotein A1 to apolipoprotein B, *B* unstandardized coefficient, *Beta* standardized coefficient

**Table 9:** Relationship between serum lipid parameters and relative factors in the males and females of the Han and Maonan populations

Lipid	Risk factor	B	Std.error	Beta	*t*	*P*
Han/male						
TC	Waist circumference	0.016	0.006	0.123	2.640	0.009
	Glucose	0.060	0.028	0.098	2.126	0.034
TG	BMI	0.006	0.003	0.111	2.073	0.037
	Weight	− 0.015	0.005	− 0.222	− 3.139	0.002
HDL-C	Weight	− 0.018	0.003	− 0.307	− 6.477	2.54E−10
	Alcohol consumption	0.102	0.031	0.158	3.327	0.001
LDL-C	Cigarette smoking	− 0.187	0.064	− 0.137	− 2.911	0.004
	BMI	0.031	0.010	0.142	3.052	0.002
	Glucose	0.057	0.024	0.114	2.420	0.016
ApoA1	Alcohol consumption	0.197	0.015	0.519	12.809	4.48E−7
	Weight	− 0.005	0.001	− 0.142	− 3.648	2.97E−4
ApoB	Waist circumference	0.007	0.001	0.262	4.837	1.83E−6
	Glucose	0.020	0.006	0.152	3.472	0.001
ApoA1/ApoB	Waist circumference	− 0.011	0.005	− 0.147	− 2.121	0.035
	Alcohol consumption	0.221	0.034	0.300	6.572	1.44E−9
Han/female						
TC	Systolic blood pressure	0.011	0.003	0.192	3.394	0.001
	Age	0.009	0.003	0.106	2.437	0.015
TG	Height	− 0.014	0.006	− 0.091	− 2.242	0.025
	Waist circumference	0.019	0.006	0.128	3.055	0.002
HDL-C	Waist circumference	− 0.011	0.003	− 0.142	− 3.563	3.95E−4
	Pulse pressure	0.002	0.001	0.109	2.803	0.005
LDL-C	Systolic blood pressure	0.005	0.002	0.107	2.566	0.011
	Waist circumference	0.018	0.005	0.149	3.745	1.91E−4
ApoA1	Cigarette smoking	0.317	0.057	0.215	5.558	4.07E−8
	Weight	− 0.005	0.001	− 0.152	− 3.907	1.04E−4
ApoB	Waist circumference	0.007	0.001	0.240	6.191	1.09E−9
	Pulse pressure	0.002	0.001	0.141	3.643	2.92E−4
ApoA1/ApoB	Waist circumference	− 0.016	0.003	− 0.202	− 5.172	3.14E−7
	Cigarette smoking	0.580	0.137	0.165	4.225	2.75E−5
Maonan/male						
TC	Waist circumference	− 0.012	0.001	− 0.276	− 8.270	5.04E−6
	Alcohol consumption	0.131	0.023	0.206	5.594	2.98E−8
TG	Waist circumference	− 0.019	0.002	− 0.311	− 9.736	2.53E−7
	Weight	0.010	0.004	0.114	2.541	0.005
	Genotype	− 0.06	0.026	− 0.073	− 2.313	0.021
HDL-C	Pulse pressure	− 0.002	0.001	− 0.078	− 2.202	0.028
	Alcohol consumption	0.057	0.048	0.113	3.801	5.23E−4
LDL-C	Weight	0.013	0.004	0.170	3.589	3.61E−4
	Alcohol consumption	0.121	0.022	0.265	5.579	4.48E−8
ApoA1	Waist circumference	− 0.007	0.002	− 0.165	− 3.486	0.001
	Glucose	− 0.026	0.012	− 0.103	− 2.163	0.031
ApoB	Alcohol consumption	− 0.029	0.011	− 0.122	− 2.670	0.008
	Age	0.001	0.001	0.095	2.000	0.046
ApoA1/ApoB	Waist circumference	− 0.021	0.003	− 0.293	− 6.435	3.56E−10
	Glucose	− 0.043	0.021	− 0.093	− 2.032	0.043
Maonan/female	Age	0.015	0.003	0.193	4.788	2.08E−6
TC	Waist circumference	0.019	0.005	0.153	3.852	1.28E−4
	Glucose	− 0.077	0.031	− 0.095	− 2.459	0.014
TG	BMI	0.156	0.049	0.122	3.183	0.002
	Alcohol consumption	0.471	0.165	0.109	2.862	0.004
HDL-C	Weight	0.002	0.001	0.093	2.676	0.008
	Alcohol consumption	− 1.066	0.152	− 0.252	− 7.014	5.68E−12
LDL-C	Waist circumference	0.022	0.003	0.242	6.552	1.13E−10
	Alcohol consumption	− 0.312	0.044	− 0.260	− 7.023	5.35E−12
ApoA1	BMI	− 0.006	0.002	− 0.100	− 2.708	0.007
	Waist circumference	0.496	0.042	0.399	11.810	2.27E−9
ApoB	Waist circumference	0.005	0.001	0.185	5.353	1.19E−7
	Alcohol consumption	− 0.687	0.101	− 0.243	− 6.786	2.54E−11
ApoA1/ApoB	Waist circumference	− 0.011	0.002	− 0.182	− 4.942	9.79E−7

The correlation among serum lipid parameters and the genotypes and several environmental factors was determined by multivariable linear regression analyses with stepwise modeling

*TC* total cholesterol, *TG* triglyceride, *HDL-C* high-density lipoprotein cholesterol, *LDL-C* low-density lipoprotein cholesterol, *ApoA1* apolipoprotein A1, *ApoB* apolipoprotein B, *ApoA1/ApoB* the ratio of apolipoprotein A1 to apolipoprotein B, *B* unstandardized coefficient, *Beta* standardized coefficient

### Relative factors for serum lipid parameters

**Fig. 5:**
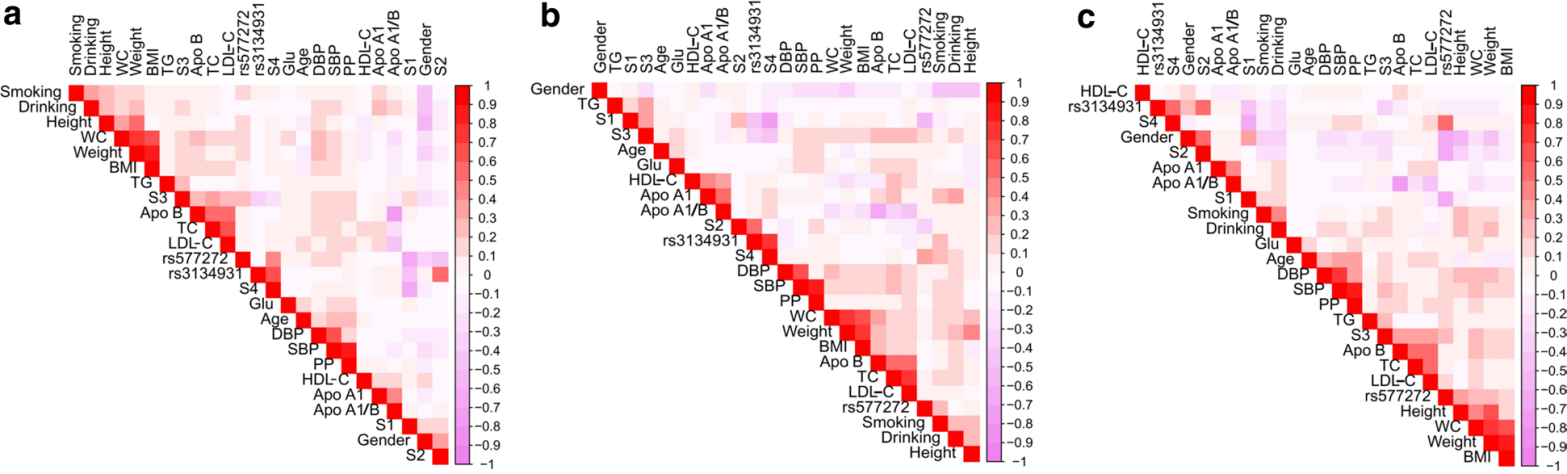
Correlations among environmental exposures and serum lipid variables, as well as the candidate loci and several haplotypes in Han + Maonan (**a**), Han (**b**) and Maonan (**c**) groups. *TC* total cholesterol, *TG* triglyceride, *HDL-C* high-density lipoprotein cholesterol, *LDL-C* low-density lipoprotein cholesterol, *ApoA1* apolipoprotein A1, *ApoB* apolipoprotein B, *ApoA1/B* the ratio of apolipoprotein A1 to apolipoprotein B, *BMI* body mass index, *Glu* glucose, *SBP* systolic blood pressure, *DBP* diastolic blood pressure, *WC* waist circumference

As shown in Fig. 5, Pearson correlation analysis suggested that the *SLC44A4* rs577272 and *NOTCH4* rs3134931 SNPs were connected with serum lipid levels. Several environmental factors such as weight, gender, height, age, waist circumference, alcohol consumption, cigarette smoking, BMI and blood pressure levels were also correlated with serum lipid parameters in both ethnic groups.

## Discussion

The main findings of the current research included the following aspects: (1) It revealed that the genotype frequencies of *SLC44A4* rs577272 and *NOTCH4* rs3134931 SNPs were significantly different between Han and Maonan populations. (2) The *SLC44A4* rs577272 SNP was associated with TC and HDL-C in Maonan ethnic group, the *NOTCH4* rs3134931 SNP was associated with TG in Han, TG and LDL-C in Maonan ethnic groups. (3) Stratified analysis according to gender showed that the *SLC44A4* rs577272 SNP was associated with TC and HDL-C in Han and Maonan females; TC in Maonan males, meanwhile, the *NOTCH4* rs3134931 SNP was associated with TG and HDL-C in Han males; TG in Han females; TG and LDL-C in Maonan males; and TG, HDL-C and LDL-C in Maonan females.

A lot of studies have showed that HLP as a severe risk factor for CHD, may be due to the combined effects of various elements, just as the age, gender, lifestyle, genetic background, environmental factors and their interactions [35, 36]. HLP acts as a highly hereditary disease, about 40–60% of the variation in serum lipid profile determined by heredity [37]. The mutation rate of *SLC44A4* rs577272 and *NOTCH4* rs3134931 SNPs was diverse amongst various origins. As per the HapMap data, the occurrence of rs577272G allele was 42.7% in Chinese, 48.3% in American, 33.5% in Italian, 35.0% in Kenyan and 34.3% in Japanese and 45.6% in European population. At the same time, the occurrence of rs3134931A allele was 47.6% in Chinese, 43.6% in Japanese, 55.3% in Yoruba, 67.6% in Italian, 53.5% in Kenyan, 57% in Mexican, and 69.5% in European population. However, the genotypic and allelic frequencies of the *SLC44A4* rs577272 and *NOTCH4* rs3134931 SNPs have not been reported previously in Maonan group. In this study, we firstly reported that the frequencies of rs577272G allele and AG, GG genotypes were 50%, 50% and 25%; rs3134931A allele and AG, AA genotypes were 46%, 49% and 22%; respectively. It means the frequencies of the rare homozygous genotype and minor allele of two selected SNPs were different between European and Asian. The above results indicated that the frequencies of minor allele or rare homozygous genotype of selected 2 SNPs would be shared a racial/ethnic-specificity. We speculated that the differences in blood lipid levels between the two ethnic groups might partly be attributed to the differences in the genotype frequencies of the two SNPs.

Previous studies suggested that plasma concentrations of TG, TC, LDL-C, HDL-C were the most important risk factors for CHD and targets for therapeutic intervention [38]. At the same time, CRP was a marker of chronic inflammation that was closely associated with CHD [39], and some clinical studies showed that a synergistic effect of statin therapy on the reducing of CRP and LDL-C, which suggested that lipids and inflammation may share some biological pathways [40, 41]. Previous studies have also identified that the *SLC44A4* rs577272 SNP is associated with serum TC and CRP levels. In addition, the *NOTCH4* rs3134931 SNP was highly associated with circulating serum or plasma MPO levels, which were responsible for the incidence as well as development of the CHD and ischemic stroke [15, 42]. At the same time, high circulating levels of MPO in serum, plasma, or white blood cells could be used as a predictor of major cardiac adverse events in healthy people and in patients with CHD or heart failure [43–46]. Furthermore, MPO has been demonstrated to be linked to some traditional risk factors that associated with CHD, just as sex, age, BMI, blood pressure, glucose, smoking and drinking habits [47–49]. MPO-derived oxidants were involved in the development of atherogenic low-density lipoprotein particles, the formation of dysfunctional HDL particles, catalytic consumption of nitric oxide, inflammatory injury of the vascular endothelium, and progression of atherosclerotic plaque and its clinical sequelae [50–52]. Although, the potential association between the *SLC44A4* rs577272, *NOTCH4* rs3134931 SNPs and blood lipid parameters was not previously documented in the Maonan population, the results of the current research clearly indicated that the levels of TC were higher and those of HDL-C were lower in the rs577272G allele carriers than in the rs577272G allele non-carriers in Maonan ethnic group. Meanwhile, the rs3134931A allele carriers had higher TG levels in Han nationality and higher TG and LDL-C levels in Maonan ethnic group than the rs3134931A allele non-carriers.

Important inter-genetic LD associations were also found in the current study. A strong linkage imbalance was detected between the two loci in both Han and Maonan ethnic groups. The haplotype of rs577272G-rs3134931A was the commonest one and accounted for more than 50% of the samples. The haplotype of the rs577272G-rs3134931A was related to an increased morbidity of HLP in the both Han and Maonan groups. At the same time, the haplotype of rs577272G-rs3134931A was associated with TG and HDL-C levels in Han; TC, TG and HDL-C levels in Maonan ethnic groups. We also noticed that haplotypes could explain more changes in serum lipid parameters than any single SNP alone particularly for TC, TG and HDL-C.

Previous studies indicated that several environmental factors were significantly associated with blood lipid spectrums, including hypertension, obesity, daily exercise, diet and lifestyle [53–56]. In the current study, we also noticed that there was association between BMI, age, blood pressure, alcohol consumption, gender, cigarette smoking and serum lipid levels in both Han and Maonan ethnic groups, suggesting that several environmental factors may also play a crucial role in influencing serum lipid levels. The marriage custom, dietary habits and lifestyle were significantly different between Han and Maonan populations. The marriage custom in Maonan is relatively conservative. Parents mainly arrange their marriages. The people of Maonan still maintain the custom of intra-ethnic marriages. Thus, intermarriage with other ethnic groups is very rare. This may be the main reason why the genetic characteristics and genotype frequencies of some lipid metabolism-related SNPs were different between the Maonan and Han populations.

Rice acts as a staple food of Maonan people. In addition, corn, potato, wheat, sorghum and so on are also be components of their diet. Maonan people especially like to eat some food that rich of oil, spicy, acid and salt. This type of diet rich in long-term high saturated fat might contribute to obesity, hypertension, high blood glucose levels, atherosclerosis and HLP [57]. Previous research has proven that the diet rich in long-term high saturated fat might contribute to a series of harmful effects on the metabolism of blood lipids, especially increased the levels of TG and TC [58]. A clinical study suggested that different doses of alcohol intake might have diverse effects on the development of atherosclerosis [59]. Several compelling researches have suggested that moderate drinking could reduce the incidence of cardiovascular events, the potential mechanism may be associated with the increased levels of HDL-C and ApoA1 [60]. However, frequent binge drinking was correlated with an increased risk of CHD mortality because it will lead to a number of serious health problems including dyslipidaemia, abnormal liver function and MI [61]. A series of recent researches also have proven that excessive drinking [57] and smoking [62, 63] were directly related to the occurrence and development of HLP. In this study, we noticed that the number of subjects who consumed alcohol and smoked were greater in Maonan than in Han groups and the number of subjects who smoked or consumed alcohol were greater in HLP than in normal groups. Thus, the combined effects of lifestyle factors, various eating habits and environmental aspects perhaps further alter the relationship of hereditary variations and serum lipid levels observed in the current research.

This study may have several limitations. To begin with, in the statistical analysis, we were not in a position to mitigate the effects of diet and some environmental factors. Secondly, other serum lipid parameters such as HDL2, small dense LDL, large buoyant LDL etc. had not been measured in our study. Thirdly, regardless of the fact that we observe a significantly correlation between the *SLC44A4* rs577272, *NOTCH4* rs3134931 SNPs and serum lipid levels, other genomic as well as environmental factors are necessary to be considered. The future studies need to be done to study the effects of either gene–gene or gene-environment or environment-environment on serum lipid levels. In order to further demonstrate our findings, some efficient studies on the natural functions of the *SLC44A4* rs577272 and *NOTCH4* rs3134931 mutations are essential.

## Conclusions

The associations of the *SLC44A4* rs577272, *NOTCH4* rs3134931 SNPs and serum lipid levels were not similar between Han and Maonan populations as well as among men and women in both ethnic groups. There might be a race- and/or gender-specific relationship of the *SLC44A4* rs577272, *NOTCH4* rs3134931 SNPs and serum lipid levels. Haplotypes could explain more changes in serum lipid parameters than any single SNP alone particularly for TC, TG and HDL-C.

## Data Availability

The datasets generated during the present study are not publicly available, because detailed genetic information of each participant were included in these materials.

## Abbreviations

ANCOVA: Covariance analysis
Apo: Apolipoprotein
BMI: Body mass index
CHD: Coronary heart disease
CRP: C-reactive protein
DNA: Deoxyribonucleic acid
GWAS: Genome-wide association study
HDL-C: High-density lipoprotein cholesterol
HLP: Hyperlipidaemia
HWE: Hardy–Weinberg equilibrium
IS: Ischemic stroke
LD: Linkage disequilibrium
LDL-C: Low-density lipoprotein cholesterol
MI: Myocardial infarction
MPO: Myeloperoxidase
NOTCH4: Neurogenic locus notch homolog protein 4
PCR: Polymerase chain reaction
RFLP: Restriction fragment length polymorphism
SLC44A4: Solute carrier family 44 member 4
SNP: Single nucleotide polymorphisms
T2DM: Type 2 diabetes mellitus
TC: Total cholesterol
TG: Triglyceride
